# BDNF-related biomarkers and cognitive impairment in chronic kidney disease: associations and machine learning-based discrimination analysis

**DOI:** 10.3389/fmed.2026.1951634

**Published:** 2026-10-21

**Authors:** Xiaoran Li, Hong Qian, Wenwen Xiao

**Affiliations:** 1 Taizhou Fourth People’s Hospital, Taizhou, Jiangsu, China; 2 College of Pharmacy, Taizhou University, Taizhou, China; 3 Eastern Theater Command Centers for Disease Control and Prevention, Nanjing, China

**Keywords:** biomarkers, brain-derived neurotrophic factor, chronic kidney disease, cognitive impairment, machine learning

## Abstract

**Background:**

Cognitive impairment is a common complication among patients with chronic kidney disease (CKD). However, biomarkers associated with cognitive impairment in this population remain insufficiently characterized. Brain-derived neurotrophic factor (BDNF) may be involved in neurobiological processes related to cognitive function, but its association with cognitive impairment among patients with CKD remains unclear.

**Methods:**

In this cross-sectional study, 240 patients with CKD underwent cognitive assessment and serum BDNF measurement during the same clinical evaluation. Multivariable logistic regression models were used to examine the associations of BDNF and the BDNF/eGFR ratio with prevalent cognitive impairment. Restricted cubic spline analyses were performed to assess potential dose–response relationships, and subgroup analyses were conducted to explore potential effect modification. Additionally, nine machine learning algorithms were applied to evaluate the discrimination ability of BDNF-related indicators for distinguishing participants with and without cognitive impairment. SHapley Additive exPlanations (SHAP) analysis was used to interpret the contribution of variables in the best-performing model.

**Results:**

Higher levels of BDNF and BDNF/eGFR ratio were significantly associated with lower odds of prevalent cognitive impairment after multivariable adjustment (BDNF: OR = 0.72, 95% CI: 0.63–0.82; BDNF/eGFR ratio: OR = 0.62, 95% CI: 0.45–0.82). Quartile analyses showed consistent inverse associations, and restricted cubic spline analyses indicated linear relationships. Significant effect modification was observed by sex for both markers and by stroke history for the BDNF/eGFR ratio. Among the evaluated machine learning approaches, the random forest model demonstrated the highest discrimination performance, with AUCs of 0.803 for BDNF and 0.844 for BDNF/eGFR ratio. SHAP analysis indicated that BDNF-related markers contributed substantially to the classification of cognitive impairment status.

**Conclusion:**

BDNF and BDNF/eGFR ratio were inversely associated with prevalent cognitive impairment in patients with CKD. These findings suggest that BDNF-related markers may be associated with cognitive impairment status and provide potential biological insights into CKD-related cognitive dysfunction. Further longitudinal studies are required to determine whether these biomarkers can contribute to future risk assessment or clinical decision-making.

## 1 Introduction

Chronic kidney disease (CKD) represents a major global public health challenge, affecting a substantial proportion of the adult population and contributing to increased morbidity and mortality worldwide (1, 2). Beyond its well-established renal and cardiovascular complications, CKD is increasingly recognized as an important risk factor for cognitive impairment (3). Cognitive dysfunction is highly prevalent among individuals with CKD, particularly in those with advanced disease, and is characterized by impairments in memory, executive function, and information processing (4, 5). CKD-related cognitive impairment is associated with poorer quality of life, reduced medication adherence, impaired self-management capacity, increased risk of hospitalization, and higher mortality. The underlying mechanisms are complex and multifactorial, involving vascular injury, chronic inflammation, oxidative stress, uremic toxin accumulation, and disruption of the kidney–brain axis (6, 7). Given the substantial clinical consequences and the potential for early intervention, timely identification and continuous monitoring of cognitive impairment in patients with CKD are of critical importance. However, effective biomarkers for early detection and risk stratification of CKD-associated cognitive decline remain limited, highlighting the need to identify novel biological indicators that can facilitate early screening and personalized management.

Brain-derived neurotrophic factor (BDNF), a key member of the neurotrophin family, plays essential roles in neuronal survival, synaptic plasticity, neurogenesis, and cognitive function. Circulating BDNF levels have been considered a potential peripheral marker of brain health, with lower BDNF concentrations associated with cognitive decline and neurodegenerative disorders (8, 9). However, its role in patients with CKD remains poorly understood. Estimated glomerular filtration rate (eGFR) is the primary indicator of kidney function and has been consistently associated with cognitive impairment, supporting the existence of a kidney–brain axis mediated by vascular injury, inflammation, oxidative stress, and uremic toxin accumulation (10–12). Nevertheless, eGFR alone reflects renal filtration capacity but does not capture individual differences in neuronal vulnerability. Given the bidirectional interactions between renal dysfunction and impaired neuroplasticity, integrating BDNF with eGFR may provide a more comprehensive indicator of CKD-related cognitive risk (13–15). To date, few studies have investigated the combined predictive value of neurotrophic biomarkers and renal function parameters for cognitive impairment in CKD populations, highlighting the need to explore novel biomarkers for early identification and risk stratification of cognitive decline.

To address these knowledge gaps, this study aimed to investigate the associations of BDNF and BDNF/eGFR ratio with prevalent cognitive impairment among patients with CKD and to evaluate their ability to discriminate between participants with and without cognitive impairment. By integrating conventional epidemiological analyses, dose–response assessment, subgroup evaluation, and machine learning-based classification approaches with explainable artificial intelligence, we sought to comprehensively characterize the relationship between BDNF-related markers and cognitive impairment status in CKD. The findings of this study may provide further insights into the potential involvement of BDNF-related pathways in the kidney–brain interaction and generate hypotheses for future longitudinal investigations evaluating the prognostic and clinical implications of these biomarkers.

## 2 Methods

### 2.1 Study population

This study was conducted at the Taizhou Fourth People's Hospital (Taizhou, Jiangsu Province, China). The study population comprised 240 patients with CKD identified from hospital records between January 2024 and January 2026 who underwent clinical evaluation and laboratory examinations at the hospital. Patients with a documented diagnosis of CKD during the study period were screened for eligibility. CKD was defined according to the Kidney Disease: Improving Global Outcomes (KDIGO) criteria as abnormalities of kidney structure or function persisting for at least 3 months and having implications for health (16). In particular, a persistent estimated glomerular filtration rate (eGFR) <60 mL/min/1.73 m^2^ was considered indicative of CKD when supported by the clinical records. CKD severity was classified according to the KDIGO GFR categories. Patients receiving dialysis were identified separately according to their documented dialysis status. Estimated glomerular filtration rate (eGFR) was calculated from serum creatinine using the 2021 Chronic Kidney Disease Epidemiology Collaboration (CKD-EPI) creatinine equation without a race coefficient. The equation was: eGFR = 142 × min(SCr/κ, 1)^α^ × max(SCr/κ, 1)^−1.200^ × 0.9938^Age^ × 1.012 (if female) (17). Patients were eligible if they met the following criteria: (1) diagnosis of CKD during hospitalization; (2) availability of demographic, clinical, cognitive assessment, and laboratory data; and (3) availability of blood samples for BDNF measurement. Patients were excluded if they had incomplete clinical information, missing cognitive assessment results, or unavailable BDNF measurements. Dialysis status was assessed based on the medical records available at the time of study inclusion. No patients in the study were receiving dialysis at enrollment. Clinical information, demographic characteristics, medical history, biochemical measurements, and cognitive function assessments were collected from the hospital electronic medical records and clinical databases. Because this was a retrospective analysis of routinely collected hospital records, no separate prospective screening log was maintained. Therefore, exact numbers of records screened and excluded at each stage could not be reliably reconstructed from the research dataset. The final analytic cohort comprised 240 patients who met the prespecified eligibility criteria and had complete data for the primary analyses.

### 2.2 Exposures

Blood samples were collected during the same clinical evaluation period as cognitive assessment. Serum was obtained after clotting at room temperature for approximately 30 min and centrifugation at 2,000 × g for 10 min, followed by aliquoting and storage at −80° C until analysis. Serum BDNF concentrations were measured using an ELISA in the clinical laboratory of hospital according to standard laboratory procedures. The primary exposures of interest were serum brain-derived neurotrophic factor (BDNF) levels and the BDNF-to-estimated glomerular filtration rate (BDNF/eGFR) ratio. The BDNF/eGFR ratio was calculated by dividing serum BDNF concentration by eGFR to reflect the relative level of neurotrophic activity in relation to renal function.

### 2.3 Covariates and outcomes

The primary outcome was cognitive impairment. Cognitive function was assessed using the Montreal Cognitive Assessment (MoCA), a standardized 30-point cognitive screening instrument covering multiple cognitive domains, including visuospatial/executive function, attention, memory, language, and orientation. The Chinese version of the MoCA has been validated in Chinese populations and has demonstrated good screening performance for cognitive impairment. The MoCA was administered and scored according to standardized procedures by clinical staff. Cognitive impairment was defined according to the prespecified MoCA cutoff of 26, based on previously validated criteria in the relevant Chinese population (18). Participants with missing or incomplete cognitive assessments were excluded from the primary analysis. Potential confounding variables were selected based on previous literature and clinical relevance. Demographic characteristics included age and sex. Clinical comorbidities included history of stroke, hypertension, and diabetes mellitus. Renal function was assessed using estimated glomerular filtration rate (eGFR). All covariates were obtained from clinical records and standardized assessments at baseline.

### 2.4 Statistical analysis

Continuous variables were presented as mean ± standard deviation (SD) for normally distributed data or as median with interquartile range (IQR) for skewed distributions. Categorical variables were summarized as frequencies and percentages. Differences between patients with and without cognitive impairment were evaluated using the independent-samples Student's *t*-test or Mann–Whitney *U* test for continuous variables, as appropriate, and the chi-square test or Fisher's exact test for categorical variables.

The associations between BDNF-related indicators and cognitive impairment were examined using multivariable logistic regression models. For analyses examining serum BDNF alone, Model 1 was adjusted for age and sex, while Model 2 additionally adjusted for stroke history, hypertension, diabetes mellitus, and eGFR. For the exploratory BDNF/eGFR ratio, the same covariates were included in Model 2 except for eGFR, because eGFR was mathematically incorporated into the exposure and further adjustment for the denominator could introduce mathematical coupling, collinearity, or difficult-to-interpret associations. The BDNF/eGFR ratio was therefore considered an exploratory composite index rather than an established clinical biomarker. BDNF and BDNF/eGFR ratio were analyzed both as continuous variables and as categorical variables according to quartiles (Q1–Q4), with the lowest quartile (Q1) used as the reference group. Odds ratios (ORs) and corresponding 95% confidence intervals (CIs) were calculated. The associations of continuous BDNF and the BDNF/eGFR ratio with cognitive impairment were reported using their respective measurement units.

Potential nonlinear associations between BDNF-related indicators and cognitive impairment were evaluated using restricted cubic spline (RCS) analyses with three knots. The presence of nonlinearity was assessed using likelihood ratio tests by comparing models with only linear terms against models incorporating spline terms.

Subgroup analyses were performed to explore potential effect modification of the associations between BDNF-related indicators and cognitive impairment. Stratified analyses were conducted according to clinically relevant variables, and interaction terms were introduced into regression models to assess statistical interactions.

For machine-learning-based classification, candidate variables included age, sex, stroke history, hypertension, diabetes, and either BDNF or the BDNF/eGFR ratio. Participants with missing values in the outcome or candidate variables were excluded using a complete-case approach. The dataset was randomly divided into a development cohort (70%) and a held-out testing cohort (30%) using a fixed random seed. Categorical variables were converted to dummy variables based on the development cohort, and continuous variables were standardized for SVM, elastic net, k-nearest neighbors, and neural network models. No class weighting, oversampling, or SMOTE was applied. Nine classification algorithms were evaluated, including generalized linear model (GLM), random forest (RF), support vector machine (SVM), elastic net (EN), k-nearest neighbors (kNN), gradient boosting machine (GBM), neural network (NNet), naïve Bayes (NB), and classification and regression tree (CART). Five-fold cross-validation within the development cohort was used for hyperparameter tuning. For caret-based models, hyperparameters were tuned using the corresponding predefined tuning grids, with a tuning length of 5 where applicable. Because of the relatively small overall sample size and the limited size of the held-out testing cohort, these analyses were considered exploratory comparisons of contemporaneous classification performance rather than formal model validation. No algorithm was designated as superior on the basis of the testing-set results. The testing-set ROC curves were used to summarize discrimination, and AUC estimates with 95% confidence intervals were reported. We did not use the testing-set ROC curve to select an optimal classification threshold, and threshold-dependent performance measures were not interpreted as evidence of clinical utility. Calibration plots were used descriptively to compare predicted and observed probabilities. Decision curve analyses, where presented, were interpreted as exploratory estimates rather than demonstrations of clinical usefulness. The best-performing model was further interpreted using SHapley Additive exPlanations (SHAP) analysis to quantify feature contributions and visualize individual and overall predictor effects.

All statistical analyses were conducted using R software (version 4.5.3). A two-sided *P* value <0.05 was considered statistically significant.

## 3 Results

### 3.1 Basic characteristics of patients

A total of 240 patients with CKD were included in the analysis. Among them, 129 patients were classified as having cognitive impairment, whereas 111 patients were classified as having no cognitive impairment. The CKD stage distribution of the participants was shown in Supplementary Table S1. As shown in Table 1, patients with cognitive impairment were significantly older than those without cognitive impairment. The prevalence of stroke history was also significantly higher in the cognitive impairment group. No significant differences were observed between the two groups in terms of sex distribution, hypertension, or diabetes prevalence. In addition, serum BDNF levels were significantly lower in patients with cognitive impairment compared with those without cognitive impairment (8.95 ± 2.27 vs. 10.71 ± 2.49 ng/mL, *P* < 0.001).

**Table 1:** Basic characteristics of the study population.

Characteristics	Overall	Non-cognitive impairment	Cognitive impairment	*P* value
*N*	240	111	129	
Age [years, mean (SD),]	49.89 (12.05)	45.77 (11.45)	53.43 (11.45)	<0.001
<60	182 (75.8)	94 (84.7)	88 (68.2)	
≥60	58 (24.2)	17 (15.3)	41 (31.8)	
Sex (*n*, %)				0.851
Male	169 (70.4)	77 (69.4)	92 (71.3)	
Female	71 (29.6)	34 (30.6)	37 (28.7)	
Stroke (*n*, %)				0.001
Yes	31 (12.9)	5 (4.5)	26 (20.2)	
No	209 (87.1)	106 (95.5)	103 (79.8)	
Hypertension (*n*, %)				1
Yes	217 (90.4)	100 (90.1)	117 (90.7)	
No	23 (9.6)	11 (9.9)	12 (9.3)	
Diabetes (*n*, %)				0.217
Yes	62 (25.8)	24 (21.6)	38 (29.5)	
No	178 (74.2)	87 (78.4)	91 (70.5)	
eGFR [mL/min/1.73 m^2^, mean (SD)]	9.01 (6.44)	7.53 (3.91)	10.28 (7.79)	0.001
BDNF [ng/mL, mean (SD)]	9.76 (2.53)	10.71 (2.49)	8.95 (2.27)	<0.001

EGFR, estimated glomerular filtration rate; brain-derived neurotrophic factor.

### 3.2 Association between BDNF-related markers and cognitive impairment in patients with CKD

The associations between BDNF-related markers and cognitive impairment are presented in Table 2. In the unadjusted model, both BDNF and BDNF/eGFR ratio were inversely associated with cognitive impairment (BDNF: OR = 0.74, 95% CI: 0.66–0.83; BDNF/eGFR: OR = 0.60, 95% CI: 0.45–0.78). After adjustment for age, sex, stroke history, hypertension, diabetes, and eGFR, these associations remained significant (BDNF: OR = 0.72, 95% CI: 0.63–0.82; BDNF/eGFR: OR = 0.62, 95% CI: 0.45–0.82).

**Table 2:** Association between BDNF-related markers and cognitive impairment in patients with CKD.

Results	Continuous OR (95% CI), per 1-unit increase	Q1	Q2	Q3	Q4	*P*-trend
BDNF			≤7.64	7.64–9.50	>9.50	
Model1	0.74 (0.66–0.83)	Ref	0.39 (0.17–0.85)	0.28 (0.12–0.60)	0.11 (0.05–0.24)	<0.001
Model2	0.72 (0.63–0.82)	Ref	0.32 (0.12–0.81)	0.26 (0.10–0.63)	0.09 (0.03–0.23)	<0.001
BDNF/eGFR			≤0.83	0.83–1.30	>1.30	
Model1	0.60 (0.45–0.78)	Ref	1.00 (0.46–2.17)	0.41 (0.19–0.85)	0.21 (0.10–0.46)	<0.001
Model2	0.62 (0.45–0.82)	Ref	1.68 (0.69–4.12)	0.55 (0.23–1.28)	0.27 (0.11–0.64)	<0.001

OR, odds ratio; Ref, reference; Q, quartile.

Quartile analyses showed consistent dose-response patterns. Compared with the lowest quartile, participants in the highest quartile of BDNF had significantly lower odds of cognitive impairment (Q4 vs. Q1: OR = 0.09, 95% CI: 0.03–0.23; *P* for trend <0.001). Similarly, higher BDNF/eGFR ratio levels were associated with reduced odds of cognitive impairment (Q4 vs. Q1: OR = 0.27, 95% CI: 0.11–0.64; *P* for trend <0.001). Spearman correlation analysis showed no significant correlation between BDNF and eGFR (*ρ* = −0.039, *P* = 0.552). In the joint model including BDNF and eGFR, all variance inflation factors were low, indicating no substantial multicollinearity (Supplementary Tables S2, S3).

### 3.3 Dose-response relationship

The potential nonlinear relationships between BDNF-related markers and cognitive impairment were assessed using RCS analyses (Figure 1). A significant dose-response relationship was observed between BDNF levels and cognitive impairment, with the odds of cognitive impairment progressively decreasing as BDNF concentrations increased. Similarly, BDNF/eGFR ratio showed an inverse dose-response association with cognitive impairment. The BDNF/eGFR ratio showed evidence of a nonlinear association with cognitive impairment (*P*-nonlinear = 0.024), while no significant evidence of nonlinearity was detected for BDNF.

**Figure 1:**
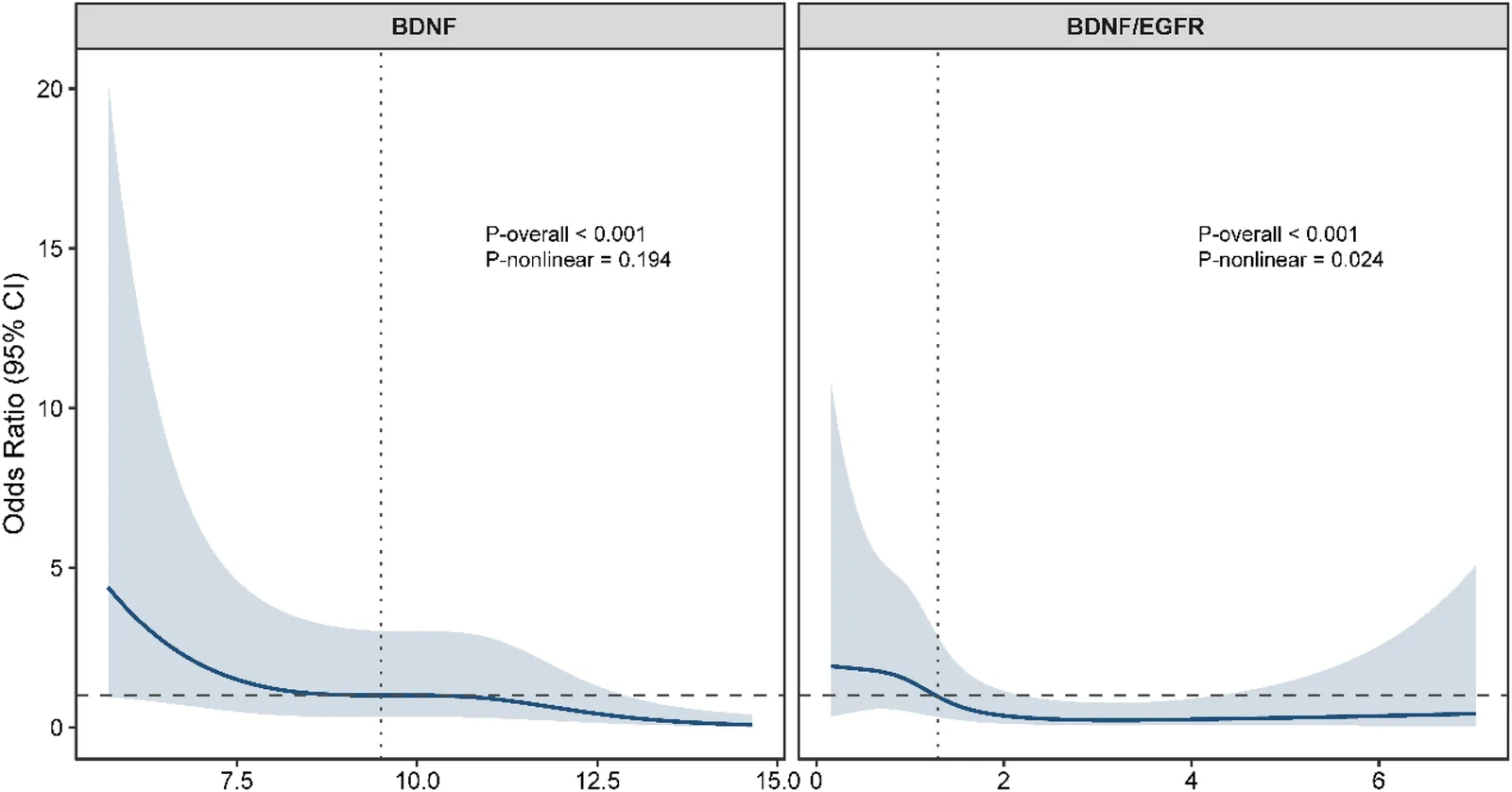
Dose-response relationships between BDNF-related indicators and cognitive impairment in patients with chronic kidney disease.

### 3.4 Subgroup analysis

Subgroup analyses were performed to evaluate the robustness and potential effect modification of the associations between BDNF-related markers and cognitive impairment across different clinical characteristics, including age, sex, stroke history, hypertension, and diabetes status (Figure 2 and Supplementary Tables S4, S5). For BDNF, significant inverse associations with cognitive impairment were observed among participants aged <60 years (OR = 0.76, 95% CI: 0.67–0.86), those aged ≥60 years (OR = 0.68, 95% CI: 0.47–0.92), females (OR = 0.84, 95% CI: 0.72–0.96), males (OR = 0.49, 95% CI: 0.32–0.68), and patients with diabetes (OR = 0.71, 95% CI: 0.61–0.81). A significant interaction was observed for sex (*P* for interaction = 0.004), suggesting that the association between BDNF and cognitive impairment differed by sex. No significant interactions were detected for age, stroke history, hypertension, or diabetes status. For BDNF/eGFR ratio, inverse associations were consistently observed across several subgroups, including younger participants (<60 years: OR = 0.60, 95% CI: 0.43–0.82), older participants (≥60 years: OR = 0.30, 95% CI: 0.10–0.69), females (OR = 0.26, 95% CI: 0.10–0.54), males (OR = 0.64, 95% CI: 0.45–0.88), and participants with stroke history (OR = 0.44, 95% CI: 0.29–0.63). Significant interactions were identified for sex (*P* for interaction = 0.037) and stroke history (*P* for interaction = 0.028). In contrast, no significant effect modification was observed for age, hypertension, or diabetes status.

**Figure 2:**
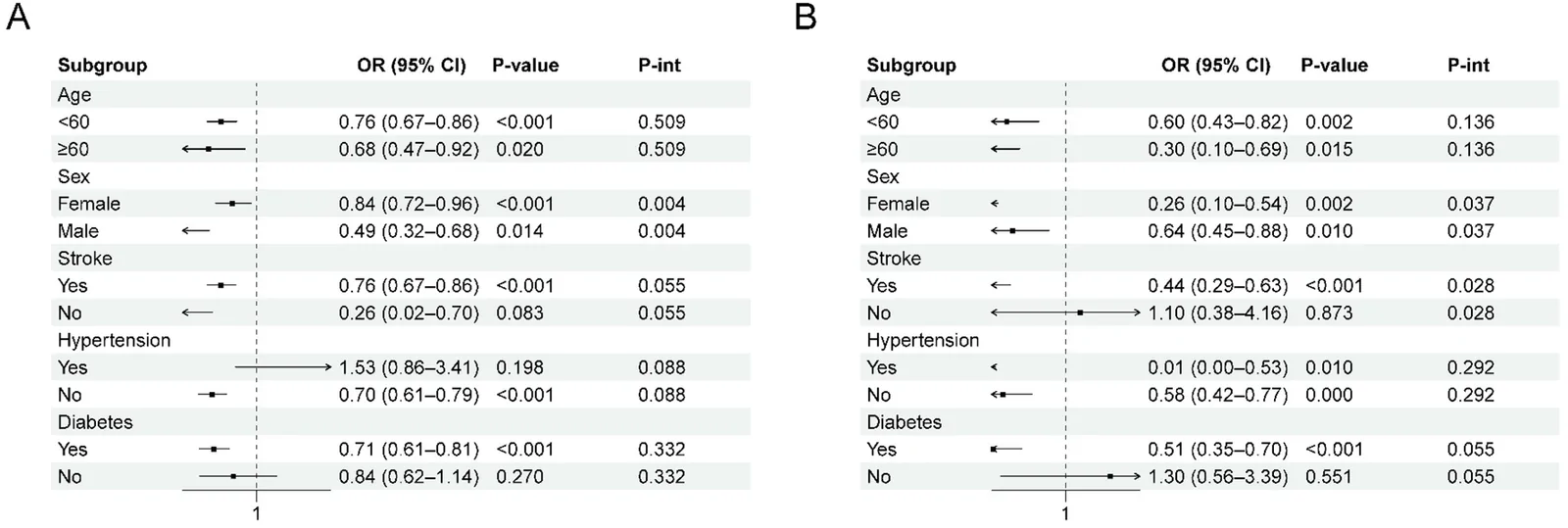
Subgroup analysis of BDNF-related indicators and cognitive function in patients with CKD. **(A)** BDNF. **(B)** BDNF/EGFR.

### 3.5 Machine learning-based classification of cognitive impairment status

Machine learning-based classification models were explored to descriptively compare the discriminatory performance of BDNF-related indicators for distinguishing participants with and without cognitive impairment among patients with CKD. The performance of models incorporating BDNF is presented in Supplementary Table S6. Across the evaluated algorithms, the RF model yielded an AUC of 0.803 (95% CI: 0.700–0.906), while the SVM model yielded an AUC of 0.801 (95% CI: 0.698–0.905). These results provide a descriptive comparison of classification performance across the evaluated algorithms rather than evidence that one algorithm was superior to the others. When the BDNF/eGFR ratio was incorporated, the observed classification performance was generally higher across the evaluated models (Supplementary Table S7). The RF model yielded an AUC of 0.844 (95% CI: 0.752–0.937), with an accuracy of 0.803, sensitivity of 0.816, specificity of 0.788, and *F*1 score of 0.816. The neural network model yielded an AUC of 0.834 (95% CI: 0.734–0.933), whereas the other evaluated algorithms showed comparatively lower AUC estimates. These findings are presented as exploratory and descriptive comparisons of model performance, without implying statistically established superiority of any individual algorithm. ROC curve analyses provided a visual comparison of discrimination across the evaluated models (Figure 3). Calibration analyses showed the observed patterns of agreement between estimated probabilities and cognitive impairment status for the evaluated models (Supplementary Figures S1–S3). Decision curve analyses illustrated differences in estimated net benefit across threshold probabilities (Supplementary Figures S2–S4). Given the cross-sectional study design, relatively small sample size, and lack of external validation, the machine learning analyses were considered exploratory classification analyses intended to describe and compare the observed performance of different algorithms. They do not establish the superiority of any specific algorithm, future cognitive risk prediction, generalizability to other populations, or clinical applicability.

**Figure 3:**
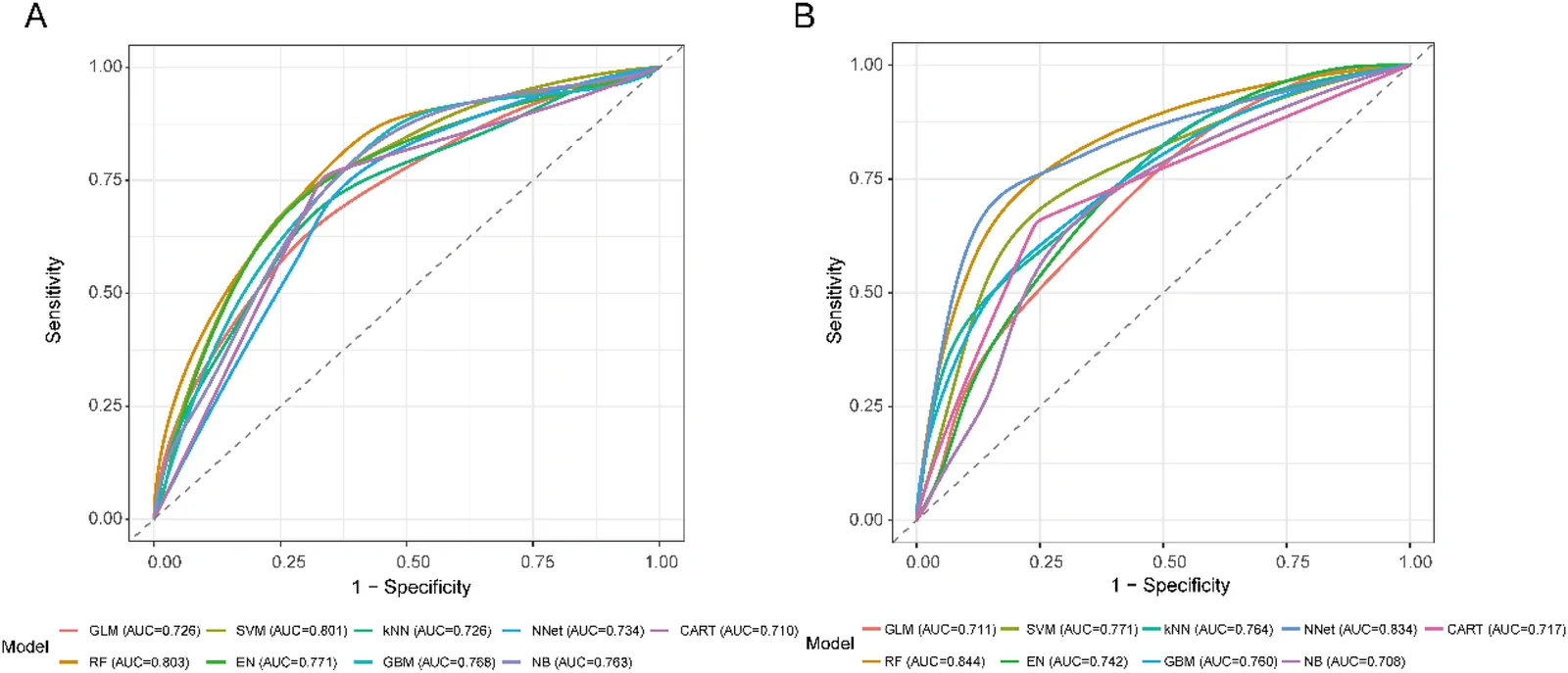
Receiver operating characteristic curves of machine learning models for predicting cognitive impairment. **(A)** BDNF. **(B)** BDNF/EGFR. ROC curves were generated for different machine learning algorithms, including generalized linear model (GLM), random forest (RF), support vector machine (SVM), elastic net (EN), k-nearest neighbors (kNN), gradient boosting machine (GBM), neural network (NNet), naïve Bayes (NB), classification and regression tree (CART). Receiver operating characteristic (ROC) curves of the machine-learning models in the held-out test cohort (*n* = 72). Models were trained and tuned in the training cohort (*n* = 168) using 5-fold cross-validation. AUCs were calculated from the original unsmoothed ROC curves, whereas smoothed curves are shown for visualization.

### 3.6 SHAP-based interpretation of machine learning classification models

Because the random forest (RF) model demonstrated the highest discrimination performance among the evaluated algorithms, SHAP analysis was applied to interpret the contribution of individual features in the RF classification models based on BDNF and BDNF/eGFR ratio (Figures 4, 5). For the BDNF-based RF model, SHAP summary and feature importance plots indicated that BDNF was one of the major contributors to the classification of cognitive impairment status, followed by other clinical characteristics. Higher BDNF values were associated with negative SHAP values, indicating a lower contribution to the classification probability of cognitive impairment, which was consistent with the inverse association observed in the regression analyses. SHAP force plots further demonstrated that the classification results were influenced by the combined contributions of BDNF and clinical characteristics, with BDNF showing an important contribution to model classification. Similarly, in the BDNF/eGFR ratio-based RF model, the BDNF/eGFR ratio showed the largest contribution among the evaluated features. Higher BDNF/eGFR ratio values were associated with lower SHAP values and reduced contribution to the classification of cognitive impairment. SHAP dependence and force plots demonstrated that the classification output reflected the combined effects of BDNF/eGFR-related information and other clinical variables, with additional features contributing to the discrimination between participants with and without cognitive impairment.

**Figure 4:**
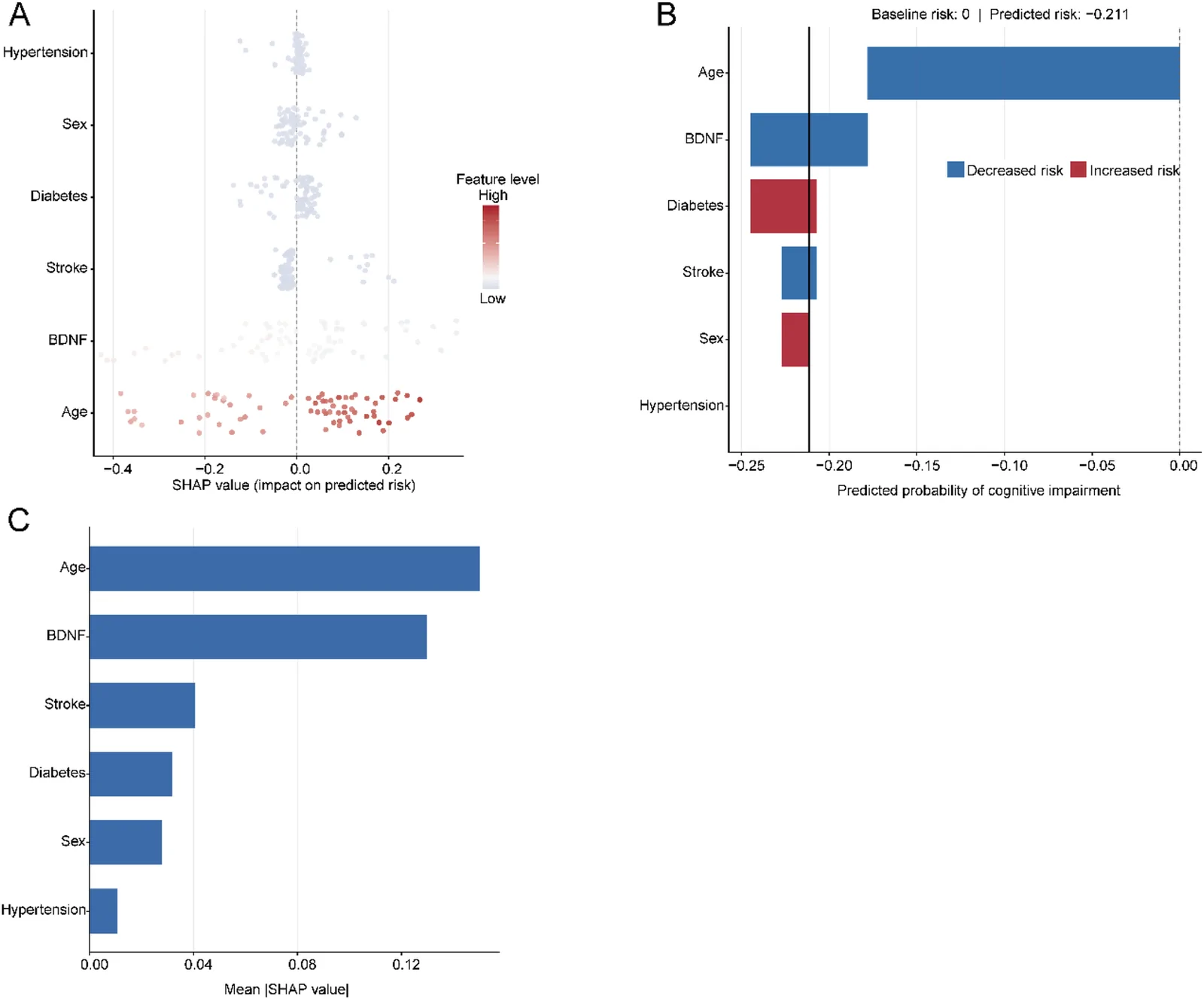
SHAP analysis of BDNF in the random forest model for cognitive impairment prediction. **(A)** SHAP summary plot. **(B)** SHAP force plot. **(C)** SHAP importance plot.

**Figure 5:**
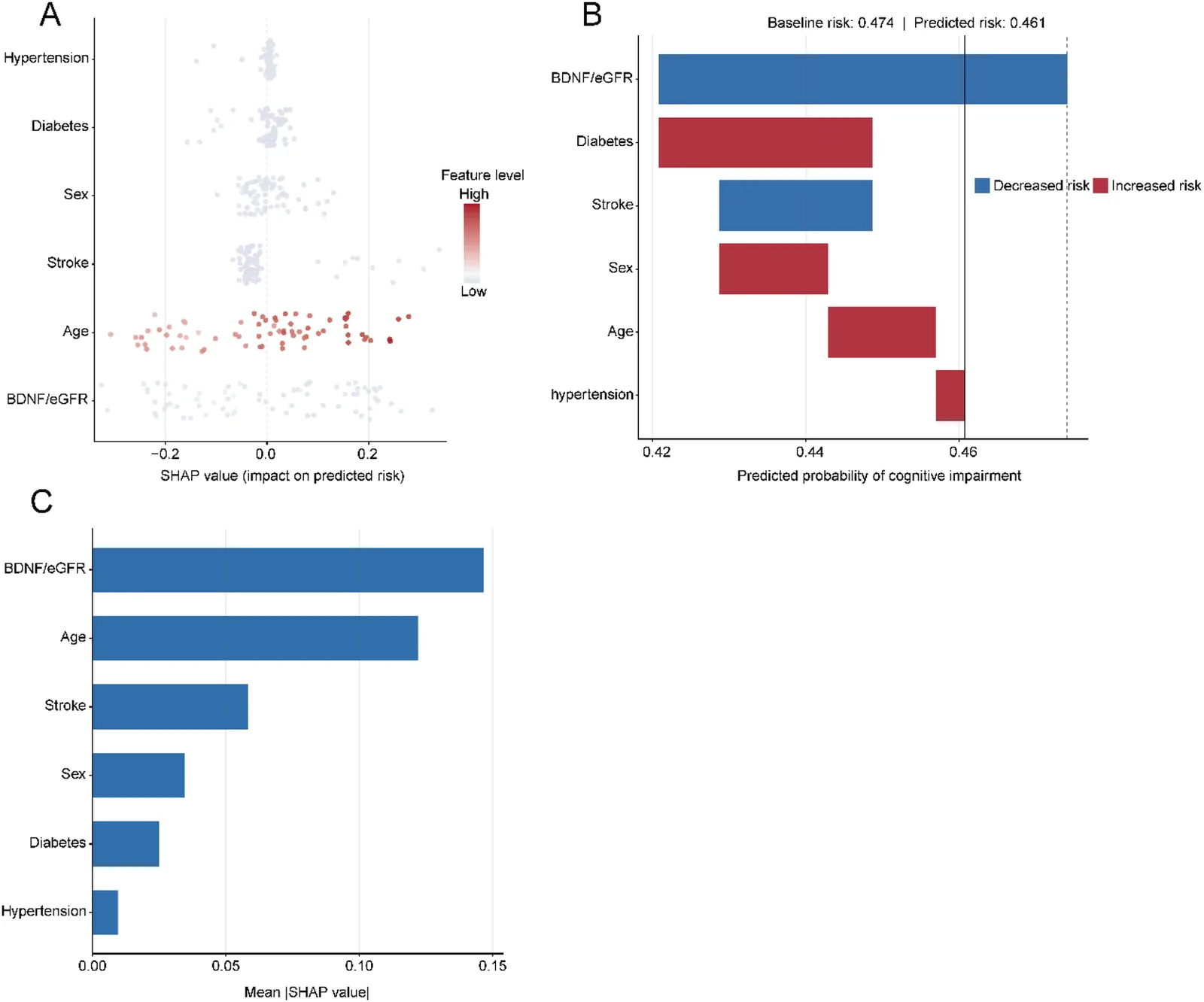
SHAP analysis of BDNF/EGFR in the random forest model for cognitive impairment prediction. **(A)** SHAP summary plot. **(B)** SHAP force plot. **(C)** SHAP importance plot.

## 4 Discussion

In this study, we systematically investigated the associations between BDNF-related markers and cognitive impairment among patients with CKD and further developed interpretable machine learning models for cognitive impairment prediction. We found that both serum BDNF levels and the BDNF/eGFR ratio were independently associated with cognitive impairment after adjustment for potential confounders. Restricted cubic spline analyses demonstrated consistent linear inverse dose-response relationships between these markers and cognitive impairment. Subgroup analyses further suggested that these associations were generally robust across clinically relevant populations, although sex and stroke history may modify the effects of BDNF-related indicators. In addition, machine learning models incorporating BDNF-related markers showed good predictive performance, with the random forest model achieving the highest discrimination, particularly when BDNF/eGFR ratio was used as the predictor. SHAP-based interpretation further confirmed the important contribution of BDNF-related indicators to model prediction and provided an interpretable framework for identifying CKD patients at higher risk of cognitive impairment. Collectively, our findings highlight the potential value of BDNF-related biomarkers as both associative indicators and predictive tools for cognitive impairment in patients with CKD.

Our findings demonstrated that both BDNF and BDNF/eGFR ratio were independently associated with lower odds of cognitive impairment in patients with CKD, showing consistent dose-response relationships after multivariable adjustment. These results are in line with previous epidemiological evidence indicating that circulating BDNF is closely related to cognitive function. As a key neurotrophic factor involved in neuronal survival, synaptic plasticity, and memory formation through the BDNF/TrkB pathway, reduced peripheral BDNF levels have been associated with cognitive decline and neurodegenerative disorders in population-based studies and meta-analyses (19). For example, a meta-analysis including 29 studies reported significantly lower peripheral BDNF levels in patients with Alzheimer's disease than in cognitively normal controls, whereas a similar decrease in individuals with mild cognitive impairment did not reach statistical significance (20). CKD may further amplify cognitive vulnerability through chronic inflammation, oxidative stress, vascular dysfunction, and accumulation of uremic toxins, which may impair BDNF-related neuroprotective pathways (21, 22). Notably, the observed association between the BDNF/eGFR ratio and cognitive impairment indicates that a combined indicator incorporating renal function and BDNF may capture complementary aspects of CKD-related physiological and neurotrophic status. However, whether the BDNF/eGFR ratio provides additional or superior information compared with BDNF alone was not formally evaluated in this study. Therefore, these findings should be regarded as exploratory and hypothesis-generating, and further studies are needed to determine whether the BDNF/eGFR ratio offers incremental value beyond BDNF alone.

Our subgroup analyses further supported the robustness of the associations between BDNF-related markers and cognitive impairment across clinically relevant populations. The inverse associations of BDNF and BDNF/eGFR ratio with cognitive impairment were generally consistent across age, sex, and metabolic comorbidity subgroups, suggesting that these biomarkers may reflect fundamental neurobiological processes underlying cognitive vulnerability in CKD rather than being restricted to specific patient characteristics. Previous studies have suggested that sex may influence circulating BDNF concentrations and its relationship with cognition, possibly due to differences in sex hormones, inflammation, and neuroplasticity regulation (23). For example, a population-based study from the Baltimore Longitudinal Study of Aging reported that serum BDNF levels and their associations with cognitive outcomes differed between men and women, highlighting potential sex-specific regulation of neurotrophic pathways (24). In our study, significant interactions were observed for sex in both BDNF and BDNF/eGFR ratio, indicating that sex-related biological differences may modify the relationship between neurotrophic status and cognitive impairment. In addition, stroke history significantly modified the association between BDNF/eGFR ratio and cognitive impairment. This finding is biologically plausible because cerebrovascular injury represents a major contributor to cognitive impairment in CKD, and impaired BDNF signaling may affect neuronal repair, synaptic remodeling, and recovery after vascular brain injury (25, 26). Previous cohort studies have demonstrated that CKD patients with cerebrovascular disease exhibit substantially higher risks of cognitive decline compared with those without vascular events, supporting the potential interaction between renal dysfunction, vascular injury, and neurotrophic impairment (27, 28). Collectively, these findings suggest that BDNF-related indicators may have broad relevance for cognitive risk assessment in CKD, while sex and cerebrovascular status may represent important factors influencing individual susceptibility.

Machine learning approaches have increasingly been applied in CKD research because cognitive impairment involves complex interactions among renal dysfunction, vascular alterations, inflammation, and neurobiological processes that may not be fully characterized by conventional regression approaches alone (29, 30). In the present study, machine learning models incorporating BDNF-related indicators demonstrated favorable discrimination performance for distinguishing participants with and without cognitive impairment. Among the evaluated algorithms, the random forest (RF) model showed the highest discrimination ability, particularly when BDNF/eGFR ratio was incorporated (AUC = 0.844, 95% CI: 0.752–0.937). Previous studies have demonstrated that machine learning algorithms can identify complex nonlinear patterns and interactions among clinical variables in cognitive research (31). For example, Na et al. developed a gradient boosting machine model to predict future cognitive impairment among community-dwelling older adults, demonstrating the potential application of artificial intelligence approaches in longitudinal cognitive studies (32). Similarly, recent studies in CKD populations have suggested that machine learning models integrating demographic, laboratory, and clinical characteristics can provide accurate prediction of adverse neurological outcomes and may facilitate individualized patient management (33, 34). The relatively higher discrimination performance of the RF model in our study may be related to its ensemble learning framework, which combines multiple decision trees and can accommodate nonlinear associations and interactions among variables. The improved discrimination observed for models incorporating the BDNF/eGFR ratio compared with BDNF alone suggests that integrating renal function-related information may provide additional information regarding the relationship between neurotrophic status and cognitive impairment in CKD. Furthermore, SHAP-based interpretation indicated that BDNF-related markers contributed substantially to the classification of cognitive impairment status, providing insights into the potential biological relevance of these markers. However, given the cross-sectional design, these machine learning models should be considered exploratory classification models rather than tools for predicting future cognitive risk or guiding clinical decision-making. Further longitudinal studies with external validation are needed to determine whether BDNF-related markers have prognostic value for subsequent cognitive changes in patients with CKD.

The application of SHAP analysis provided additional interpretability for the machine learning models and helped characterize the contribution of BDNF-related indicators to the classification of cognitive impairment status (35, 36). Although machine learning algorithms can capture complex nonlinear relationships and interactions among variables, their interpretation remains challenging. SHAP, based on cooperative game theory, quantifies the contribution of individual features to model outputs and has been increasingly used to improve the interpretability of biomedical machine learning analyses (37). In our study, SHAP analyses demonstrated that BDNF and the BDNF/eGFR ratio contributed substantially to the classification outputs of the corresponding random forest models, with higher values generally associated with lower SHAP values and a reduced contribution toward the classification of cognitive impairment. These patterns were directionally consistent with the regression analyses, providing complementary information regarding the contribution of these markers within the classification models. However, SHAP feature importance reflects the contribution of variables to model classification and should not be interpreted as evidence of biological importance, independent causal effects, or mechanistic relationships. Previous studies have suggested that interpretable machine learning approaches can improve transparency and facilitate the understanding of complex biomedical models (38, 39). In our analysis, the relatively large SHAP contribution of the BDNF/eGFR ratio indicates that this variable contributed to the predictions generated by the fitted classification model within the study population. However, SHAP values reflect the contribution of variables to model predictions and do not establish independent associations, biological relevance, underlying mechanisms, or causal effects. The potential prognostic or clinical relevance of the BDNF/eGFR ratio therefore requires evaluation in larger longitudinal cohorts with independent validation. Overall, the SHAP analyses were used to improve the interpretability of the machine learning models by illustrating the relative contribution of BDNF-related variables to the model-generated classifications of cognitive impairment status. These findings should be considered exploratory and hypothesis-generating and should not be interpreted as evidence of biological mechanisms, future cognitive risk prediction, or clinical utility.

This study provides evidence of associations between BDNF-related markers and cognitive impairment in patients with CKD. We examined both BDNF and the kidney function-adjusted BDNF/eGFR ratio in relation to cognitive impairment using multivariable regression, dose–response, and subgroup analyses. The observed inverse associations may support the hypothesis that BDNF-related pathways are relevant to cognitive impairment in CKD, although the underlying mechanisms cannot be determined from the present cross-sectional data. Machine-learning and SHAP analyses further characterized the contribution of BDNF-related markers to the classification of contemporaneous cognitive impairment. These findings provide a basis for further investigation of the relationship between BDNF-related markers, kidney dysfunction, and cognitive impairment in longitudinal and mechanistic studies.

Several limitations should be acknowledged. First, the cross-sectional design limits the ability to establish temporal or causal relationships between BDNF-related markers and cognitive impairment. Because BDNF, eGFR, covariates, and cognitive status were assessed at the same time point, our findings reflect associations with contemporaneous cognitive impairment and do not establish whether BDNF-related changes precede subsequent cognitive decline. Longitudinal studies are therefore needed to clarify temporal relationships. Second, because this study was based on routinely collected hospital records, the availability of covariates was limited. Although we adjusted for several major demographic and clinical factors, potentially relevant variables, including medication use, depressive symptoms, education, socioeconomic status, nutritional status, physical activity, inflammatory markers, anemia-related parameters, detailed CKD severity, and vascular burden, were not comprehensively available and therefore could not be fully accounted for. Some of these factors may act as potential confounders, whereas others may lie on pathways linking CKD, BDNF-related markers, and cognitive impairment; therefore, additional adjustment may not always be appropriate without establishing their temporal and causal relationships. Residual confounding consequently cannot be completely excluded. Third, cognitive function was assessed using standardized screening tools rather than comprehensive neuropsychological evaluations, which may have resulted in some degree of misclassification. Fourth, the relatively limited sample size may have reduced statistical power for subgroup analyses and limited the precision and generalizability of the machine-learning models. Although SHAP analysis improved model interpretability, the machine-learning analyses should be regarded as exploratory internal model development and require validation in larger, independent CKD cohorts. The decision curve analysis provides estimated net benefit across threshold probabilities but does not establish clinical usefulness in the absence of external validation and prespecified consequences of false-positive and false-negative classifications. Fifth, the blood samples were obtained during hospitalization as part of routine clinical care, the time of day varied among participants. Although previous studies have reported inconsistent findings regarding diurnal variation in peripheral BDNF, including sex-dependent variation in plasma BDNF and changes in serum BDNF during sleep, the potential influence of sampling time cannot be completely excluded. Therefore, some variability in measured serum BDNF concentrations may have been introduced by differences in sampling time. Another limitation is that dialysis status was assessed only at the time of study enrollment. None of the participants were receiving dialysis at enrollment; however, some patients may have initiated dialysis during subsequent clinical management. Because longitudinal changes in dialysis status were not systematically available in the retrospective dataset, we could not evaluate whether dialysis initiation or maintenance was associated with subsequent changes in BDNF levels or cognitive function. In addition, although all participants had CKD at study enrollment, CKD severity may influence both BDNF-related markers and cognitive function. Our study focused on the association between BDNF-related markers and cognitive impairment within the overall CKD population rather than on differences across CKD stages. Therefore, detailed stage-specific analyses were not performed, and heterogeneity related to CKD severity may have contributed to residual confounding. Future studies with larger samples should examine whether these associations vary across different stages of CKD.

## 5 Conclusions

In conclusion, this study demonstrated that higher levels of BDNF and BDNF/eGFR ratio were independently associated with a lower risk of cognitive impairment among patients with CKD. The observed dose–response relationships and consistent findings across multiple analyses suggest that BDNF-related markers may reflect neurobiological vulnerability underlying CKD-associated cognitive decline. Furthermore, integrating BDNF-related indicators into machine learning models, particularly the random forest algorithm, showed promising predictive performance, and SHAP analysis confirmed the substantial contribution of these markers to cognitive risk prediction. These findings highlight the potential value of BDNF and BDNF/eGFR ratio as clinically relevant biomarkers for early identification and individualized risk stratification of cognitive impairment in CKD patients.

## Data Availability

The raw data supporting the conclusions of this article will be made available by the authors, without undue reservation.
