# Supplementary material for: BDNF-related biomarkers and cognitive impairment in chronic kidney disease: associations and machine learning-based discrimination analysis
Source: Front Med (Lausanne). 2026 Oct 21;13:1951634. doi: 10.3389/fmed.2026.1951634 (PMC13730932; doi:10.3389/fmed.2026.1951634)
Supplement: Supplementary file 1 [file Datasheet1.pdf]

**Supplementary material**

# BDNF-related biomarkers and cognitive impairment in chronic kidney disease: associations and machine learning-based prediction

Xiaoran Li [a 1 *], Hong Qian [b 1], Wenwen Xiao [c]

a. Taizhou Fourth People's Hospital, No. 99, Gulou Road, Hailing District, Taizhou City, Jiangsu Province, China

b. College of Pharmacy, Taizhou University, Taizhou, China

c. Eastern Theater Command Centers for Disease Control and Prevention, Nanjing, China

[1] These authors contributed equally.

[*]**Corresponding Author:**

Xiaoran Li, Taizhou Fourth People's Hospital, No. 99, Gulou Road, Hailing District, Taizhou City, Jiangsu Province, China;

**E-mail addresses:** 18262342956@163.com (Xiaoran Li)

Table S1. CKD stage distribution of the participants.

| **CKD stage** | **N (%)** |
|---|---|
| G1 | 0 (0%) |
| G2 | 0 (0%) |
| G3a | 0 (0%) |
| G3b | 6 (2.5%) |
| G4 | 18 (7.5%) |
| G5 | 216 (90%) |

Table S2. Spearman correlation between BDNF and eGFR.

| Method | Correlation | P value | N |
| --- | --- | --- | --- |
| Spearman | -0.04 | 0.55 | 240 |

Table S3. Multicollinearity diagnostics for the multivariable logistic regression model including BDNF and eGFR

| **Variable** | **VIF** |
|---|---|
| BDNF | 1.07 |
| eGFR | 1.07 |
| Age | 1.19 |
| Sex | 1.08 |
| Stroke | 1.08 |
| Hypertension | 1.05 |
| Diabetes | 1.16 |

Table S4. Subgroup analysis of BDNF and cognitive function in patients with CKD.

| Subgroup | OR (95% CI) | P-value | P-int |
|---|---|---|---|
| Age | | | |
| <60 | 0.76 (0.67–0.86) | <0.001 | 0.509 |
| >=60 | 0.68 (0.47–0.92) | 0.020 | |
| Sex | | | |
| Female | 0.84 (0.72–0.96) | <0.001 | 0.004 |
| Male | 0.49 (0.32–0.68) | 0.014 | |
| Stroke | | | |
| Yes | 0.76 (0.67–0.86) | <0.001 | 0.055 |
| No | 0.26 (0.02–0.70) | 0.083 | |
| Hypertension | | | |
| Yes | 1.53 (0.86–3.41) | 0.198 | 0.088 |
| No | 0.70 (0.61–0.79) | <0.001 | |
| Diabetes | | | |
| Yes | 0.71 (0.61–0.81) | <0.001 | 0.332 |
| No | 0.84 (0.62–1.14) | 0.270 | |

Table S5. Subgroup analysis of BDNF/EGFR and cognitive function in patients with CKD.

| Subgroup | OR (95% CI) | P-value | P-int |
|---|---|---|---|
| Age | | | |
| <60 | 0.60 (0.43–0.82) | 0.002 | 0.136 |
| >=60 | 0.30 (0.10–0.69) | 0.015 | |
| Sex | | | |
| Female | 0.26 (0.10–0.54) | 0.002 | 0.037 |
| Male | 0.64 (0.45–0.88) | 0.010 | |
| Stroke | | | |
| Yes | 0.44 (0.29–0.63) | <0.001 | 0.028 |
| No | 1.10 (0.38–4.16) | 0.873 | |
| Hypertension | | | |
| Yes | 0.01 (0.00–0.53) | 0.010 | 0.292 |
| No | 0.58 (0.42–0.77) | <0.001 | |
| Diabetes | | | |
| Yes | 0.51 (0.35–0.70) | <0.001 | 0.055 |
| No | 1.30 (0.56–3.39) | 0.551 | |

Table S6. Metrics of machine learning models in predicting BDNF and cognitive impairment.

| Model | AUC (95% CI) | Cutoff | Accuracy | Sensitivity | Specificity | PPV | NPV | F1 | TP | TN | FP | FN |
|---|---|---|---|---|---|---|---|---|---|---|---|---|
| RF | 0.803 (0.700–0.906) | 0.537 | 0.761 | 0.868 | 0.636 | 0.733 | 0.808 | 0.795 | 33 | 21 | 12 | 5 |
| SVM | 0.801 (0.698–0.905) | 0.573 | 0.761 | 0.789 | 0.727 | 0.769 | 0.750 | 0.779 | 30 | 24 | 9 | 8 |
| EN | 0.771 (0.658–0.884) | 0.556 | 0.761 | 0.711 | 0.818 | 0.818 | 0.711 | 0.761 | 27 | 27 | 6 | 11 |
| GBM | 0.768 (0.654–0.882) | 0.609 | 0.746 | 0.711 | 0.788 | 0.794 | 0.703 | 0.750 | 27 | 26 | 7 | 11 |
| NB | 0.763 (0.647–0.880) | 0.401 | 0.761 | 0.789 | 0.727 | 0.769 | 0.750 | 0.779 | 30 | 24 | 9 | 8 |
| NNet | 0.734 (0.616–0.852) | 0.456 | 0.718 | 0.763 | 0.667 | 0.725 | 0.710 | 0.744 | 29 | 22 | 11 | 9 |
| GLM | 0.726 (0.609–0.844) | 0.560 | 0.704 | 0.684 | 0.727 | 0.743 | 0.667 | 0.712 | 26 | 24 | 9 | 12 |
| kNN | 0.726 (0.609–0.843) | 0.500 | 0.718 | 0.737 | 0.697 | 0.737 | 0.697 | 0.737 | 28 | 23 | 10 | 10 |
| CART | 0.710 (0.599–0.821) | 0.556 | 0.718 | 0.763 | 0.667 | 0.725 | 0.710 | 0.744 | 29 | 22 | 11 | 9 |

Table S7. Metrics of machine learning models in predicting BDNF/eGFR and cognitive impairment.

| Model | AUC (95% CI) | Cutoff | Accuracy | Sensitivity | Specificity | PPV | NPV | F1 | TP | TN | FP | FN |
|---|---|---|---|---|---|---|---|---|---|---|---|---|
| RF | 0.844 (0.752–0.937) | 0.405 | 0.803 | 0.816 | 0.788 | 0.816 | 0.788 | 0.816 | 31 | 26 | 7 | 7 |
| NNet | 0.834 (0.734–0.933) | 0.854 | 0.817 | 0.737 | 0.909 | 0.903 | 0.750 | 0.812 | 28 | 30 | 3 | 10 |
| SVM | 0.771 (0.658–0.884) | 0.559 | 0.761 | 0.737 | 0.788 | 0.800 | 0.722 | 0.767 | 28 | 26 | 7 | 10 |
| kNN | 0.764 (0.656–0.872) | 0.700 | 0.690 | 0.474 | 0.939 | 0.900 | 0.608 | 0.621 | 18 | 31 | 2 | 20 |
| GBM | 0.760 (0.649–0.871) | 0.554 | 0.704 | 0.579 | 0.848 | 0.815 | 0.636 | 0.677 | 22 | 28 | 5 | 16 |
| EN | 0.742 (0.624–0.861) | 0.498 | 0.718 | 0.763 | 0.667 | 0.725 | 0.710 | 0.744 | 29 | 22 | 11 | 9 |
| CART | 0.717 (0.610–0.824) | 0.509 | 0.704 | 0.658 | 0.758 | 0.758 | 0.658 | 0.704 | 25 | 25 | 8 | 13 |
| GLM | 0.711 (0.589–0.834) | 0.402 | 0.704 | 0.895 | 0.485 | 0.667 | 0.800 | 0.764 | 34 | 16 | 17 | 4 |
| NB | 0.708 (0.583–0.834) | 0.299 | 0.718 | 0.737 | 0.697 | 0.737 | 0.697 | 0.737 | 28 | 23 | 10 | 10 |

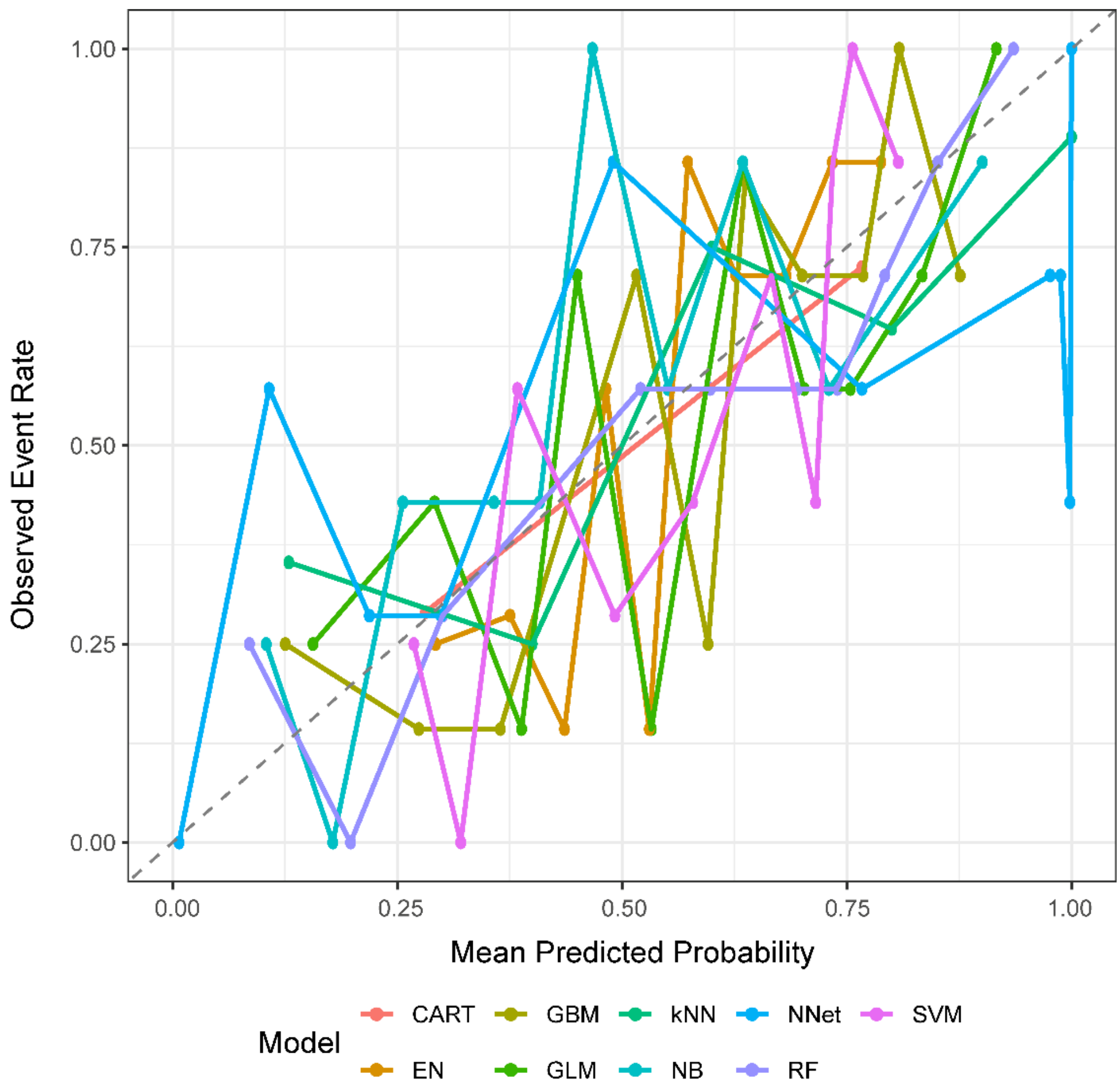


Fig. S1. Calibration curves of machine learning models for predicting cognitive impairment based on BDNF.

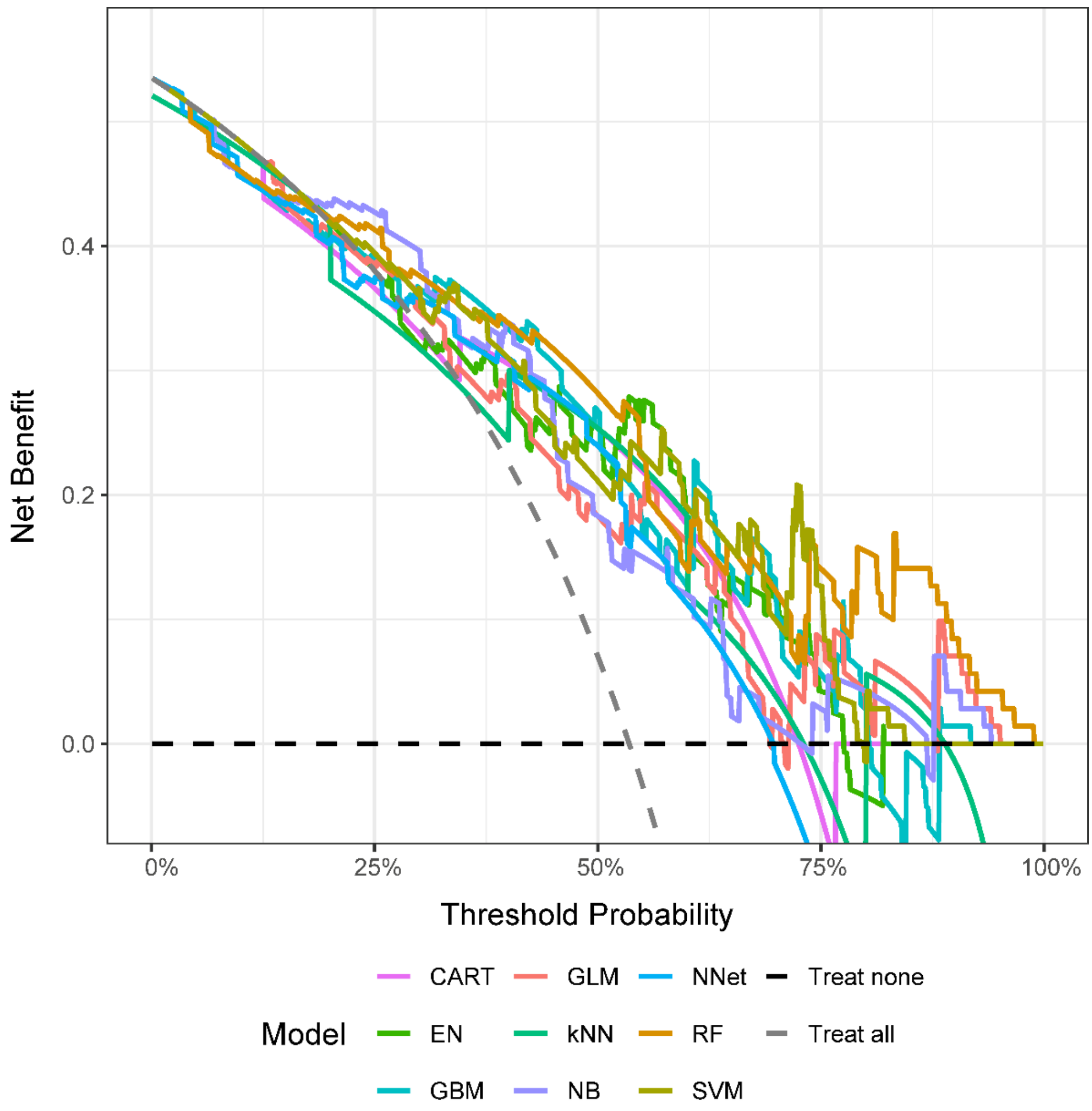


Fig. S2. Decision curve analysis (DCA) of machine learning models for predicting cognitive impairment based on BDNF.

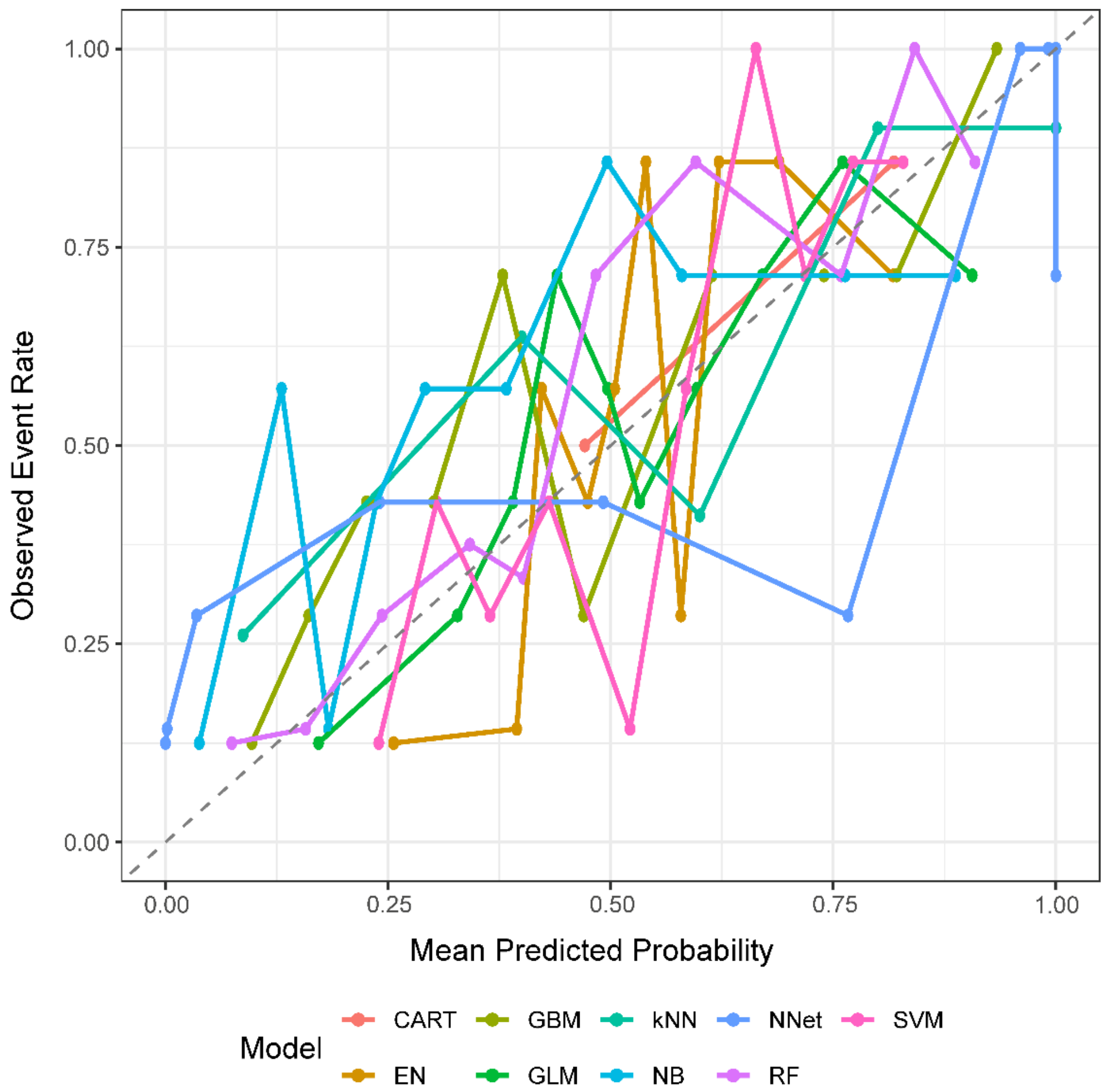


Fig. S3. Calibration curves of machine learning models for predicting cognitive impairment based on BDNF/ eGFR.

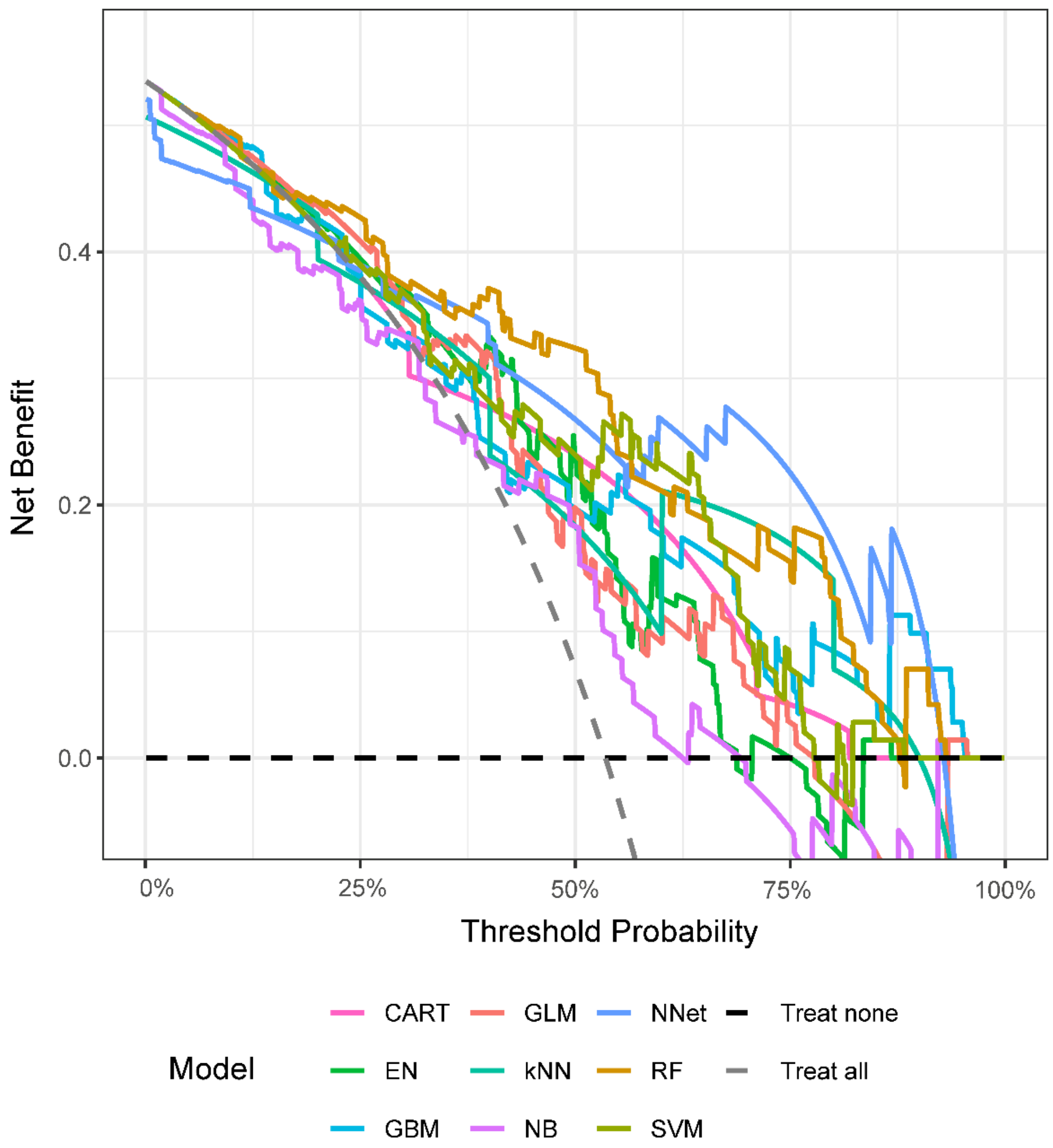


Fig. S4. Decision curve analysis (DCA) of machine learning models for predicting cognitive impairment based on BDNF/ eGFR.
